# Validation of an ultrasensitive digital droplet PCR assay for HIV-2 plasma RNA quantification

**DOI:** 10.7448/IAS.17.4.19675

**Published:** 2014-11-02

**Authors:** Jean Ruelle, Vasilieios Yfantis, Armelle Duquenne, Patrick Goubau

**Affiliations:** AIDS Reference Laboratory, Université Catholique de Louvain, Brussels, Belgium

## Abstract

**Introduction:**

Low or undetectable plasma viral load (VL) using current qPCR assays is common for HIV-2 patients. Digital PCR is an emerging technology enabling more precision and reproducibility than qPCR at low DNA/RNA copy numbers. Available data related to digital droplet PCR (ddPCR, Bio-Rad) underscore issues linked to the threshold definition of positivity, coupled to the specificity of low copy results [1].

**Materials and Methods:**

A RT-PCR protocol was set up using the One-Step RT-ddPCR Kit for Probes on the QX200 platform (Bio-Rad, Hercules, CA) in an accredited environment (ISO15189:2012 norm). Parameters tested were in line with the digital MIQE guidelines [2]. Inter-run coefficient of variation (CV) was established using synthetic RNA controls diluted in HIV-negative plasma. The ddPCR assay was compared to a qRT-PCR previously used in routine (LOQ 50 cop/mL [3]) using 46 clinical samples and the NIBSC international HIV-2 RNA standard.

**Results:**

The optimal PCR efficiency and the best separation between positive and negative droplets were obtained with a mixture containing 0.5 mM manganese acetate, 700 nM primers and 250 nM of the 5'FAM-probe. Using a manual threshold to define positivity, 7.74% of negative controls (n=168) were scored as positive due to one positive droplet. The presence of two positive droplets or more was not observed for negative controls. Serial dilutions of a positive control showed excellent linearity (R2=0.999) and enabled us to define a limit of quantification of two positives droplets, which corresponds to 0.14 copies/μL in the reaction mixture and to seven copies per mL of plasma. The inter-run coefficient of variation was 3.37% at a mean value of 4,468 cop/mL, 19.59% at 416 cop/mL and 32.28% at 8 cop/mL. The NIBSC standard of 1,000 IU was quantified 1,400 copies by ddPCR and close to 5,000 copies by qPCR (delta log superior to 0.5). Among 46 clinical samples, 22 were undetectable with both qPCR and ddPCR, 12 were detected with both methods (respective means of 10,612 and 2,224 cop/mL, delta log=0.68) and 12 others were quantified by ddPCR only below 50 cop/mL (mean=16 cop/mL).

**Conclusions:**

We validated a ddPCR HIV-2 VL assay that is more sensitive and more reproducible than the qPCR assay used as comparator, with a limit of quantification of 7 cop/mL of plasma. A careful definition of the limit of blank allows the management of false positive droplets, but the variable user-defined positive threshold may be an issue for compliance to the quality norms.
